# The Critical Influence of Wire Diameter and Bending for Orthodontic Wire Integration—New Insights for Maxillary Movements (In Vitro Study)

**DOI:** 10.3390/dj12120399

**Published:** 2024-12-06

**Authors:** Michael Moncher, Ahmed Othman, Benedikt Schneider, Fady Fahim, Constantin von See

**Affiliations:** 1 Research Center for Digital Technologies in Dentistry and CAD/CAM, Department of Dentistry, Faculty of Medicine and Dentistry, Danube Private University, Steiner Landstraße 124, 3500 Krems an der Donau, Austria; ahmed.othman@dp-uni.ac.at (A.O.); constantin.see@dp-uni.ac.at (C.v.S.); 2 Center for Oral and Maxillofacial Surgery, Department of Dentistry, Faculty of Medicine and Dentistry, Danube Private University, Steiner Landstraße 124, 3500 Krems an der Donau, Austria; benedikt.schneider@mailbox.org; 3 Orthodontic Department, Faculty of Dentistry, Cairo University, 11-ElL-Sara St. Manial, Cairo 11553, Egypt; fady.hussein@dentistry.cu.edu.eg

**Keywords:** dentistry, orthodontics, maxillary expansion, aligner therapy, orthodontic wires, biomechanics, orthodontic treatment, orthodontic tooth movement, stainless steel

## Abstract

**Background**: Traditional methods for palatal expansion using fixed appliances often face limitations in comfort and aesthetics. In comparison, aligner therapy has limitations, particularly regarding maxillary expansion. The aim of this study is to examine the biomechanical properties regarding the wire diameter and bending of different stainless steel wires to evaluate their potential for incorporation into maxillary aligner therapy. **Materials and Methods**: Three rectangular stainless steel wires (0.016″ × 0.022″, 0.017″ × 0.025″, and 0.019″ × 0.025″) were tested for mechanical expansion forces in the intermolar region, comparing non-tooth-shaped bent wires (A groups) and tooth-shaped bent wires (B groups). Using a Z010 testing machine (ZwickRoell GmbH and Co. KG, Ulm, Germany), expansion forces were measured at 1 mm intervals over a 5 mm distance, with 15 samples analyzed per group. Statistical analyses included the Shapiro–Wilk test for normal distribution, the Mann–Whitney U test, which revealed significant results (U = 225, *p* < 0.001), and the Kruskal–Wallis test, which indicated significance (H = 39.130; df = 2; *p* < 0.001). **Results**: Tooth-shaped bent wires exhibited significantly lower expansion forces than non-tooth-shaped bent wires for all tested wire types. This difference was most notable in wires with larger transverse profiles (0.019″ × 0.025″), where the tooth-shaped bent wires displayed a marked reduction in mechanical load capacity. Specific force measurements for non-tooth-shaped wires ranged from 760.61 ± 79.51 mN at 1 mm of deformation to 2468.46 ± 66.27 mN at 5 mm of deformation, while tooth-shaped wires ranged from 116.80 ± 3.74 mN to 1979.49 ± 23.23 mN. **Conclusions**: These findings suggest that non-tooth-shaped bent wires offer a more efficient and uniform expansion potential for maxillary movements due to their stable elastic properties. Clinically, integrating non-tooth-shaped stainless steel wires into aligner therapy may provide a viable method for maxillary expansion, supporting both first- and second-order movements in orthodontic treatment. Further research is needed to explore the integration of such wires for effective maxillary expansion in aligner therapy.

## 1. Introduction

Throughout the years, orthodontic treatment has undergone significant developments, driven by a continuous quest for innovative techniques to tackle various malocclusions and enhance patient outcomes. One such popular modality is aligner therapy, which is renowned for its aesthetic appeal, comfort, and convenience [1]. However, aligner therapy has its limitations, particularly in cases necessitating maxillary or intermolar expansion, and it is crucial for addressing issues such as posterior crossbites and crowding [2]. These limitations include reduced efficacy in generating sufficient expansion forces and maintaining stability for long-term results, especially in the upper jaw. Traditional aligners struggle to achieve the level of intermaxillary expansion that is often required for the comprehensive correction of transverse discrepancies in the maxilla. They may have limitations in treating complex malocclusions that require rotations of more than 25 degrees or maxillary horizontal expansion movements, where traditional braces are generally more effective [3].

While traditional methods such as palatal expanders have been effective for maxillary expansion, they may not align with all patient preferences and lifestyles [4]. Fixed functional appliances such as Hyrax or hybrid Hyrax, utilizing various anchorage methods, have established themselves as primary means for achieving maxillary expansion [5], offering better control and predictability [6].

Recent research indicates that aligner therapy can effectively aid intra-arch expansion, correcting dentoalveolar crossbites and modifying arch shape [7]. Even in mixed dentition, an aligner system can serve as a gradual maxillary expansion technique [8]. Moreover, comparative studies favor clear aligners over fixed appliances such as the quad helix for expansion, as fixed appliances impact the alveolar bone integrity, which is not the case with clear aligners [9].

Furthermore, fixed functional appliances are often rejected due to aesthetic concerns and potential adverse effects on periodontal and conservative tooth structures, including increased risk of dental caries, gingivitis, or periodontitis [10].

Against this backdrop, the present research aims to explore the biomechanical principles, such as the wire diameter and bending, of rectangular stainless steel orthodontic wires. It is hypothesized that the integration of rectangular stainless steel wires into orthodontic applications, particularly in aligner therapy, provides increased and more consistent expansion forces [11], enabling effective intermaxillary expansion that cannot be achieved with traditional aligners alone. The use of these specialized wires is intended to achieve a controllable and effective expansion that not only broadens treatment options but is also tailored to the individual anatomical circumstances and needs of patients.

Therefore, the hypothesis of this study is that the integration of stainless steel wires into orthodontic applications may improve their efficiency, particularly regarding stability and force transmission. Exploring the biomechanical principles of steel wires lays the groundwork for further study regarding the advantages, challenges, and potential benefits of combining orthodontic wires with aligners [12]. This innovative approach could address the limitations of traditional aligner therapy for maxillary expansion by enhancing stability and force application through the added wire component [13].

## 2. Materials and Methods

In this study, the following test and auxiliary devices were used for the in vitro approach to evaluating rectangular orthodontic wires for integration into aligners. To test the effectiveness of the wires in aligner therapy, stainless steel wires (Forestadent Bernhard Förster GmbH, Pforzheim, Germany) of the same length were used to evaluate forces. Stainless steel wires were chosen because they offer the highest stiffness among orthodontic wire materials, allowing them to exert significantly stronger forces over extended periods. A ZwickRoell testing machine, model Z010 10 kN ProLine (ZwickRoell GmbH and Co. KG, Ulm, Germany), which is versatile and capable of measuring static forces, was employed in this process. To measure static forces under 100 N, the load cell Xforce HP (ZwickRoell GmbH and Co. KG, Ulm, Germany) with a maximum nominal force (F_nom_) of 100 N was used. The load cell has an accuracy of 0.2 N at 0.2% of F_nom_ and an accuracy of 1.0 N at 1% of F_nom_ [14]. The testing machine was calibrated according to the DIN EN ISO 7500-1—2018 standard [15].

In the experiment, three orthodontic steel wires with different cross-sections (Wire I with 0.016″ × 0.022″, II. with 0.017″ × 0.025″, and III. with 0.019″ × 0.025″) (Forestadent Bernhard Förster GmbH, Pforzheim, Germany) were used. These three cross-sections were chosen because they are among the most frequently used in orthodontic applications. Their prevalence in clinical practice makes them relevant for evaluating the potential integration with aligners, as they represent standard dimensions in orthodontic treatments. All steel wires have a rectangular transverse profile. In addition, a rectangular cross-section minimizes the risk of shifting or twisting of the sample during testing because of the increased surface area in contact with the test stamp.

**Figure 1:**
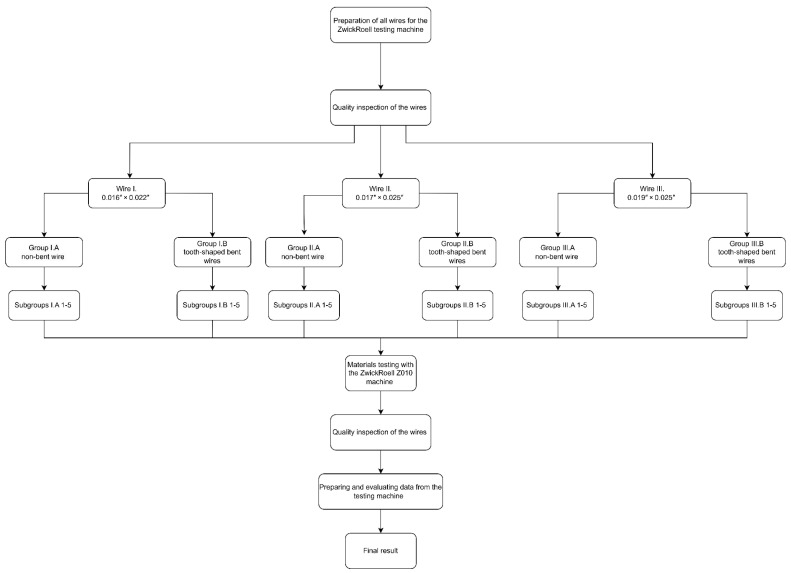
The entire experimental workflow is visualized in this flowchart.

Two independent main groups (A and B) were tested for all wire types, where all cross-sections were examined once with a non-tooth-shaped bent wire (A) group and with a tooth-shaped bent wire (B) group. The main groups were further divided into five subgroups according to the wire types (see Figure 1; I.A/B 1-5, IIA/B 1-5, III.A/B 1-5), representing the elastic range in 1 mm increments up to 5 mm. A maximum distance of 5 mm was used because, in preliminary testing, some samples exhibited plastic deformation beyond 5 mm and were no longer in the elastic phase. The first experiment was conducted with wire type I. from the main group A. The five subgroups (groups I.A1 to I.A5) were extracted based on the Excel tables generated by the testing machine. This procedure was also applied to wire type II. and wire type III. for the main groups II.A and III.A. Subsequently, the main group B (tooth-shaped bent wires) was tested following the same scheme. A total of 15 identical samples were tested per subgroup to ensure statistical reliability and account for any potential variability in the wire material or bending process. The sample size of 15 was chosen because this number has proven to be effective in preliminary studies to ensure reliable and meaningful results. The results of the preliminary studies carried out supported the decision in favor of 15 samples, as they showed sufficient statistical power and consistency to confirm the reliability of the results. This resulted in 75 samples for each main group and 150 samples for each wire cross-section. The testing machine (ZwickRoell GmbH and Co. KG, Ulm, Germany) was operated in a displacement-controlled mode to ensure the consistent deformation of the wires. All relevant parameters, such as force and displacement, were reset to zero before each test to ensure accurate and reproducible measurements.

A flowchart was created for the purpose of visualizing the experimental workflow (Figure 1).

### 2.1. Manufacture of Tooth-Shaped Bent Wires

**Figure 2:**
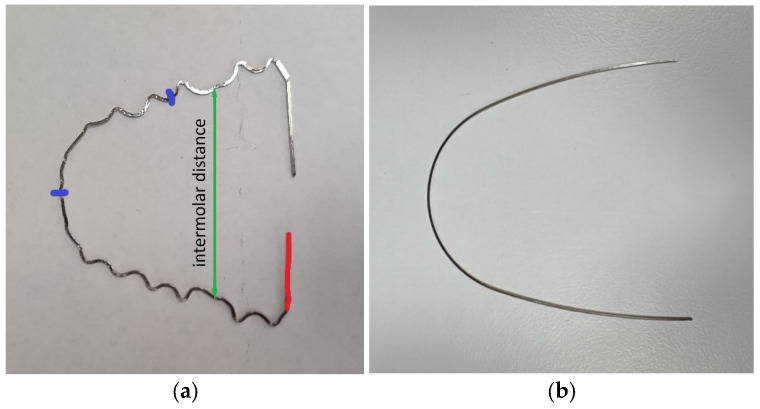
(**a**) Left side: Here, one can see a tooth-shaped bent wire. The green arrow indicates the intermolar distance, the red mark shows the side to be clamped, and the blue markers serve as orientation points for precise, repeatable clamping. (**b**) Right side: Here, one can see a non-tooth-shaped bent wire for comparison.

To fabricate the pre-bent orthodontic wires (Forestadent Bernhard Förster GmbH, Pforzheim, Germany) for material testing, 3D-printed models of an upper dental arch were produced using a digital light processing (DLP) printer (Model XS, BEGO Medical GmbH, Bremen, Germany). After completing the models, the wires were shaped with great precision and care into an identical form to ensure consistent and uniform bending for all samples. Each wire was manually bent following the same pattern and specifications, with the bending being performed exclusively in two dimensions within the horizontal plane. All wires were bent by the same certified dental technician with more than 10 years of experience, ensuring consistency and accuracy across all samples. A pre-bent reference wire was used, and all fabricated wires were aligned to the reference wire. This ensured that all wires were nearly identical in their geometric alignment and curvature. This approach was used in order to ensure that the physical properties and response to stress for each wire were as identical as possible, which was crucial for the comparability of the test results (Figure 2).

### 2.2. Experimental Setup

**Figure 3:**
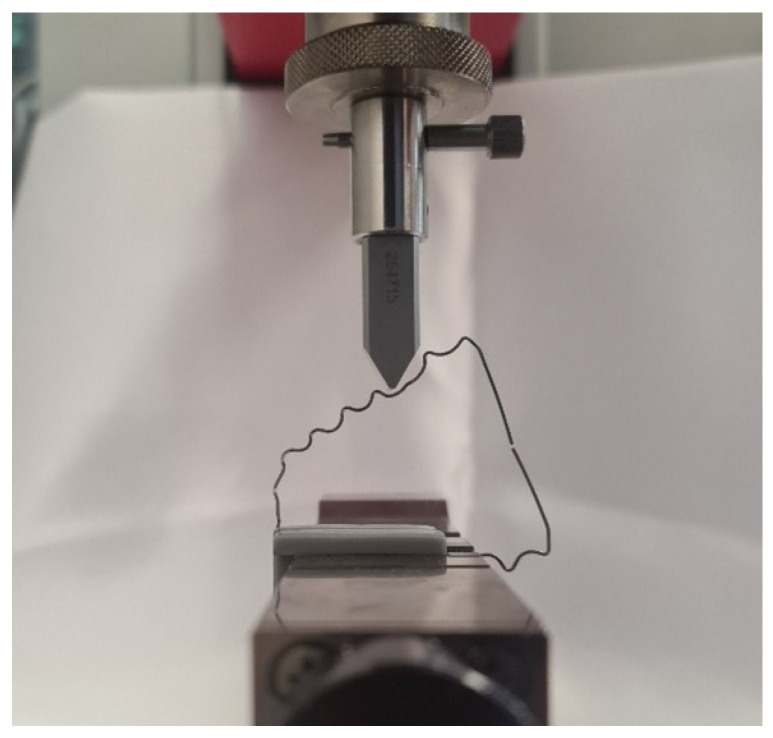
The ZwickRoell machine testing a correctly clamped tooth-shaped bent wire.

For the setup of the experiment, a clamping jaw chuck was mounted in the material testing machine to clamp the wire with the two clamping jaws (Figure 3). Above the clamping jaw chuck, a wedge-shaped test stamp was positioned and fixed with two positioning rods to prevent the wire from slipping to the left or right. This was performed identically for the wires, which were only rotated 90° to prevent slipping forward or backward.

### 2.3. Experimental Procedure

At the outset of the experiment, the steel wire was positioned on a flat surface to confirm its level and parallelism. Subsequently, the intermolar distance was gauged with a ruler to ascertain a uniform and accurate distance of 4 cm [16]. A red mark was made on the test side of the wire, as shown in Figure 1, to test the same half of the tooth-shaped bent wire in all samples. The two blue marks served for the precise positioning of the clamping jaws on the wire. In Figure 1, a blue linear marker is also visible, which was used to align the clamping jaws in the clamping jaw chuck of the material testing machine.

After positioning the jaws on the wire, they were inserted into the clamping device. The wire was aligned at exactly 90° to the feed axis. The test stamp was then lowered until it almost touched the test specimen (Figure 3). The testing machine was started with a feed rate of 1 mm/min. The wire was tested over a distance of exactly 5 mm, as this was within the elastic deformation range of the wire, allowing it to return to its original position after the test. Up to 5 mm, the compressive force of the orthodontic wire was measured with the load cell. The distance was measured in millimeters (mm), and the force was measured in millinewtons (mN).

### 2.4. Statistical Analysis

Statistical analysis was performed using SigmaPlot (Version 13, Systat Software Inc., San Jose, CA, USA). All data were tested for normal distribution using the Shapiro–Wilk test, which indicated that the results were not normally distributed. Due to the lack of a normal distribution, the Mann–Whitney U test was used to compare the A groups (non-tooth-shaped bent wires) with the B (tooth-shaped bent wires) groups. Additionally, the comparison of the different wire cross-section types (Wires I., II., III.) within the A groups and the B groups was conducted using the Kruskal–Wallis test, as this is a non-parametric method suited for comparing more than two independent groups without assuming a normal distribution.

### 2.5. Software and Tools

For the entire analysis, SigmaPlot (Version 13, Systat Software Inc., San Jose, CA, USA) and the Python programming language [17] were primarily used, specifically with the libraries Pandas (Version 1.5.3, NumFOCUS Inc., Austin, TX, USA) for data analysis, Matplotlib 3.6.2 [18] and Seaborn 0.12.1 for visualization [19], and SciPy 1.9.3 [20] for conducting statistical tests.

## 3. Results

For statistical analysis, the biomechanical properties of the tooth-shaped bent wires were examined in comparison with non-tooth-shaped bent wires. Force values were measured for three different wire cross-sections (Wires I., II., and III.) and deformation distances (1 mm to 5 mm). The results were reported as the median ± interquartile range (IQR) in millinewtons (mN).

Wire I:

For the Wire I cross-section, the force values increased with greater deformation distance in both groups. However, the tooth-shaped bent wires (group B) consistently exhibited lower force values than the non-tooth-shaped bent wires (group A).

At 1 mm of deformation:
  Group I.A1 (non-tooth-shaped): 760.61 ± 79.51 mN
  Group I.B1 (tooth-shaped): 116.80 ± 3.74 mN

At 2 mm of deformation:
  Group I.A2: 1356.18 ± 12.52 mN
  Group I.B2: 478.24 ± 15.28 mN

At 3 mm of deformation:
  Group I.A3: 1841.08 ± 43.12 mN
  Group I.B3: 1014.41 ± 30.19 mN

At 4 mm of deformation:
  Group I.A4: 2240.43 ± 41.63 mN
  Group I.B4: 1580.49 ± 12.88 mN

At 5 mm of deformation:
  Group I.A5: 2468.46 ± 66.27 mN
  Group I.B5: 1979.49 ± 23.23 mN

Wire II:

For the Wire II cross-section, a similar trend was observed, with lower force values in the tooth-shaped bent wire groups than in the non-tooth-shaped groups.

At 1 mm of deformation:
  Group II.A1: 771.48 ± 60.46 mN
  Group II.B1: 144.44 ± 11.31 mN

At 2 mm of deformation:
  Group II.A2: 1757.19 ± 87.77 mN
  Group II.B2: 784.79 ± 38.41 mN

At 3 mm of deformation:
  Group II.A3: 2586.63 ± 78.23 mN
  Group II.B3: 1483.01 ± 40.30 mN

At 4 mm of deformation:
  Group II.A4: 3227.54 ± 95.63 mN
  Group II.B4: 2007.18 ± 28.19 mN

At 5 mm of deformation:
  Group II.A5: 3711.43 ± 73.12 mN
  Group II.B5: 2400.38 ± 45.07 mN

Wire III:

For the Wire III cross-section, lower force values were also observed in the tooth-shaped bent wire groups than in the non-tooth-shaped groups.

At 1 mm of deformation:
  Group III.A1: 974.84 ± 10.26 mN
  Group III.B1: 143.56 ± 32.00 mN

At 2 mm of deformation:
  Group III.A2: 2719.78 ± 21.99 mN
  Group III.B2: 927.00 ± 84.38 mN

At 3 mm of deformation:
  Group III.A3: 4326.36 ± 24.32 mN
  Group III.B3: 1740.99 ± 57.30 mN

At 4 mm of deformation:
  Group III.A4: 5530.21 ± 28.78 mN
  Group III.B4: 2359.86 ± 51.66 mN

At 5 mm of deformation:
  Group III.A5: 6585.03 ± 90.45 mN
  Group III.B5: 2814.31 ± 50.72 mN

The Mann–Whitney U test yielded a U-value of 225 in all groups and a *p*-value ranging from 3.38334 × 10^−6^ to 3.39182 × 10^−6^. These results indicate that the differences between groups A and B are statistically significant (*p* < 0.001), regardless of the wire cross-section used and the deformation distance covered.

Furthermore, the A subgroups with different wire transverse profiles were compared with each other, as were the corresponding B subgroups. Due to the lack of a normal distribution and the presence of three different wire cross-sections, a Kruskal–Wallis one-way analysis of variance on ranks was performed. The analysis yielded an H-value of 39.130 with two degrees of freedom. This shows that the differences in median values between the wire groups (I., II., and III.) are greater than would be expected by chance; there is a statistically significant difference (*p* < 0.001) (Table 1, Table 2 and Table 3).

To summarize, the findings indicate that the force values increase in conjunction with the extent of the deformation distance in both main groups (A and B). However, group B exhibits significantly lower force values than the corresponding group A across all tested wire types and deformation distances. The statistical analysis using the Mann–Whitney U test confirmed that these differences are highly significant (U = 225, *p* < 0.001).

**Figure 4:**
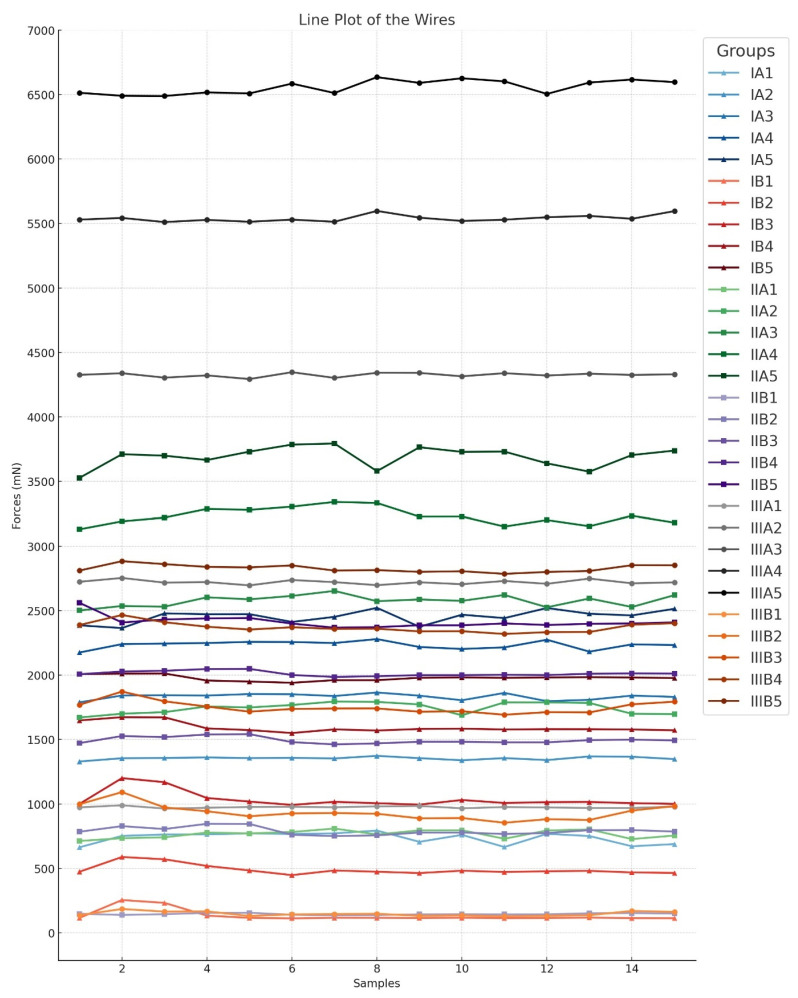
The above graph displays the results of the tests conducted. The groups with Wire I are marked with triangles, the groups with Wire II are marked with squares, and the groups with Wire III are marked with circles. Group A (non-tooth-shaped bent wires) and group B (tooth-shaped bent wires) are represented by the same color tones. The Y-axis displays the force in millinewtons (mN), while the X-axis shows the number of measured subgroups.

The comparison of the different wire types (Wires I., II., and III.) within the A groups and the B groups using the Kruskal–Wallis test also showed significant differences between the groups (H = 39.130; df = 2; *p* < 0.001). To isolate the group or groups that differ from the others, a multiple comparison procedure was performed. All pairwise multiple comparison procedures were conducted using the Tukey test (*p* < 0.050). A line plot was constructed for the purpose of visualizing the data (Figure 4) (Table 4).

## 4. Discussion

The results show promising biomechanical properties for rectangular stainless steel wires, with potential for integration into maxillary aligner therapy. Notably, the unbent wire with the largest cross-section (Wire III., group A5) achieved the best results in terms of force application, making it the most suitable for maxillary expansion. All tested wires were able to produce consistent and controlled expansion forces, indicating their suitability for supporting orthodontic applications, particularly as an adjunct to aligner therapy. These findings thus confirm the initial hypothesis that stainless steel wires can significantly increase expansion force and stability in aligner therapy.

Mechanical expansion forces in the intermolar region were examined with regard to the influence of different wire sizes and bending types on the potential effectiveness of stainless steel wires in this context. The results indicate that the differences between the different wire cross-sections and shapes are statistically significant in most cases. Notably, the tooth-shaped bent wire (B) groups consistently exhibited lower force values across all tested cross-sections compared with the unbent (A) groups. This suggests that manual adjustment of the wires plays a significant role in reducing the forces exerted on the teeth.

However, the precise manual bending of the wires presents challenges. While aiming to ensure high conformity and repeatability, manual adjustment could induce stresses in the material that alter the biomechanical properties of the wires and potentially impair the efficiency of the orthodontic treatment [21]. These additional stresses could lead to increased variability in measurement results, as observed in all B groups, indicating that manual bending does not always preserve the material properties originally specified by the manufacturer [22].

In light of this, it appears more advantageous to use unbent wires for aligner therapy and focus on the natural elastic properties of the wires. This approach could achieve the necessary expansion forces without plastic deformation from manual bending, which might lead to higher consistency of the results and reduce the risk of material fatigue and stress concentrations.

The results showed that the use of non-tooth-shaped bent wires (A) resulted in a significant improvement in expansion in the molar region compared with tooth-shaped bent wires (B). In the latter, significantly weaker forces occurred due to plastic deformation, leading to the loss of advantageous mechanical properties. The elastic phase of the non-tooth-shaped bent wire (A) groups proved particularly beneficial, allowing for uniform and constant forces to be exerted on the molars. These findings are consistent with previous studies indicating that aligners alone offer limited possibilities for maxillary expansion, especially in the molar region, where reinforcement with wires can be advantageous [7].

Aligners are increasingly being used as an alternative to conventional orthodontic treatment because they offer improved aesthetics, comfort, and the convenience of removability [23]. In recent years, the use of aligners has become increasingly popular due to their aesthetic appeal and patient comfort [24]. The concept of integrating stainless steel wires into aligner therapy could be effective by allowing controlled and constant forces on the molars without the need for additional mechanical activation tools such as screws or keys. This might simplify clinical use, offer greater comfort to patients, and reduce the necessity for extra devices. A study [25] showed that using aligners alone often leads to buccal tipping of the premolars, whereas the integration of wires in the present study allows for horizontal expansion without significant tooth tipping.

These results underscore the possible advantages of combining aligners and non-tooth-shaped bent wires for first-order movements (bucco-lingual and labio-lingual), as well as second-order movements (mesio-distal movements) [26].

Our results indicate that integrating rectangular stainless steel wires into aligner therapy could be an effective method for achieving controlled and consistent maxillary expansion. The cross-sectional area of the wire plays a significant role in the expansion force achieved. It was observed that the plastic deformation of the wires led to a marked reduction in their stability, which significantly impacted the forces acting on the molars and the expansive effect. These observations contradict the assumption that forces remain constant even after plastic deformation [27]. Instead, utilizing the elastic properties of the wires appears to be more important for maintaining the desired expansion forces.

Both the tooth-shaped bent wire (A) groups and non-tooth-shaped bent wire (B) groups are mechanically well suited for dental orthodontic corrections [4,28] but are not suitable for skeletal bone expansions [29]. To increase the acting forces, additional auxiliary devices such as springs should be used in future studies [30].

To address the limitations of this study, it must be mentioned that wire bending was performed manually by an experienced individual. Manual processing may increase variability in force values, even though the same individual performed the bendings. The use of a wire-bending machine could address this issue by ensuring consistent and reproducible forces. However, to our knowledge no wire-bending machine is currently available that can perform such small and intricate radii bending.

Another limitation of this report is the inability to determine how consistent the force exerted by stainless steel remains over time. Further studies are recommended to evaluate the long-term stability of the material’s force properties.

An additional limitation is related to the integration of stainless steel wires into orthodontic devices. The optimal method to incorporate these wires into orthodontic systems to maximize their effectiveness remains unclear, requiring further investigation to fully explore their potential applications. Furthermore, the current study is limited regarding strong clinical considerations and can only provide fundamentals and directions for further research.

In the initial considerations for further research, we propose the following two variants. The first variant involves integrating a steel wire into the aligner, with the wire being fixed palatally in the upper anterior region. In the premolar and molar areas, palatal recesses (slots) should be present in the aligner, allowing the wire to exert forces in its elastic phase on the desired positions. To generate this expansion force, a modified bracket should be attached palatally to both sides of the molars but not bonded to the tooth.

The second variant proposes not integrating the wire into an aligner but attaching it palatally like a retainer to the front teeth. As in variant one, a modified bracket is fixed palatally on both sides of the molars to exert an expansion force on the molars using the elastic phase of the wire. However, variant two has the disadvantage of being a fixed solution.

Future studies are necessary to further investigate the stability of expansion through the integration of orthodontic wires with additional methods of enforcement. Additionally, the difference between tipping movements and pure horizontal expansion should be thoroughly examined to ensure optimal treatment outcomes for different patients. Moreover, it would be interesting to conduct additional tests encompassing other characteristics such as flexural strength [31] and fatigue [32] to complete the overview of the present report. The growing preference for aligners over traditional braces underscores the need for optimization, especially in aligner-based therapies. Aligners offer a discreet and comfortable option for patients, leading to higher acceptance rates and compliance.

In future innovations, there may be ways to integrate thin steel wires or metallic components directly into the design of clear aligners themselves. For example, aligners could be designed with a reinforced metal structure that adds strength and stability to the aligner. This could potentially help in cases where the aligner needs to provide more significant force without compromising comfort or aesthetics. However, integrating steel wires into aligner therapy could present several challenges, including reduced comfort due to the added rigidity of the wires, and aesthetic concerns, as the metal components might compromise the discreet appearance of clear aligners. Additionally, the need for more customized designs could increase treatment complexity, potentially leading to longer treatment times. These factors could further impact patient compliance, as the bulkier aligners may be less comfortable and less desirable to wear consistently. Steel wires could be integrated into aligners palatally during the vacuum forming process by embedding them into the material, allowing for added structural support and force delivery without compromising the aligner’s fit. This method would enable the aligners to maintain their aesthetic appeal while incorporating the benefits of the steel wires for more controlled tooth movement in complex cases.

## 5. Conclusions

The findings of this study provide a fundamental biomechanical overview of rectangular orthodontic stainless steel wires. The biomechanical tests conducted demonstrated that tooth-shaped bent wires tend to exhibit lower expansion forces than unbent wires, suggesting that manual adjustment of the wires can reduce the mechanical load capacity. To ensure more precise and consistent expansion, unbent wires should be preferred, as they offer more stable results. Unbent wires with larger cross-sections may have potential for cases requiring higher expansion force and force stability, such as maxillary expansion. Clinically, the investigated wires could be used for first-order movements (bucco-lingual and labio-lingual), as well as second-order movements (mesio-distal movements). The selection of the optimal wire and its configuration should be based on the specific treatment scenario and individual patient needs. Future research should focus on methods for the effective incorporation of such wires into aligner splints, as well as their long-term stability and effects on orthodontic treatment. In addition, virtual treatment planning using the proposed ways of integrating wires into aligners should be investigated in vitro.

Finally, to utilize the presented wires for controlled maxillary expansion, further biomechanical and clinical research is required.

## Figures and Tables

**Table 1:** Wire I: This group was analyzed using the Mann–Whitney U test with a significance level of *p* < 0.001.

Wire I. (0.016″ × 0.022″)							
	Median Group I.A	Median ± IQR	Median Group I.B	Median ± IQR	U-Value	*p*-Value	Interpretation
Group A1 vs. Group B1	760.61	760.61 ± 79.51	116.80	116.80 ± 3.74	225	3.39182 × 10^−6^	significant
Group A2 vs. Group B2	1356.18	1356.18 ± 12.52	478.24	478.24 ± 15.28	225	3.39182 × 10^−6^	significant
Group A3 vs. Group B3	1841.08	1841.08 ± 43.12	1014.41	1014.41 ± 30.19	225	3.39182 × 10^−6^	significant
Group A4 vs. Group B4	2240.43	2240.43 ± 41.63	1580.49	1580.49 ± 12.88	225	3.39182 × 10^−6^	significant
Group A5 vs. Group B5	2468.46	2468.46 ± 66.27	1979.49	1979.49 ± 23.23	225	3.39182 × 10^−6^	significant

**Table 2:** Wire II: This group was analyzed using the Mann–Whitney U test with a significance level of *p* < 0.001.

Wire II. (0.017″ × 0.025″)							
	Median Group II.A	Median ± IQR	Median Group II.B	Median ± IQR	U-Value	*p*-Value	Interpretation
Group A1 vs. Group B1	771.48	771.48 ± 60.46	144.44	144.44 ± 11.31	225	3.39182 × 10^−6^	significant
Group A2 vs. Group B2	1757.19	1757.19 ± 87.77	784.79	784.79 ± 38.41	225	3.39182 × 10^−6^	significant
Group A3 vs. Group B3	2586.63	2586.63 ± 78.23	1483.01	1483.01 ± 40.30	225	3.39182 × 10^−6^	significant
Group A4 vs. Group B4	3227.54	3227.54 ± 95.63	2007.18	2007.18 ± 28.19	225	3.38334 × 10^−6^	significant
Group A5 vs. Group B5	3711.43	3711.43 ± 73.12	2400.38	2400.38 ± 45.07	225	3.39182 × 10^−6^	significant

**Table 3:** Wire III: This group was analyzed using the Mann–Whitney U test with a significance level of *p* < 0.001.

Wire III. (0.019″ × 0.025″)							
	Median Group III.A	Median ± IQR	Median Group III.B	Median ± IQR	U-Value	*p*-Value	Interpretation
Group A1 vs. Group B1	974.84	974.84 ± 10.26	143.56	143.56 ± 32.00	225	3.39182 × 10^−6^	significant
Group A2 vs. Group B2	2719.78	2719.78 ± 21.99	927.00	927.00 ± 84.38	225	3.39182 × 10^−6^	significant
Group A3 vs. Group B3	4326.36	4326.36 ± 24.32	1740.99	1740.99 ± 57.30	225	3.39182 × 10^−6^	significant
Group A4 vs. Group B4	5530.21	5530.21 ± 28.78	2359.86	2359.86 ± 51.66	225	3.38334 × 10^−6^	significant
Group A5 vs. Group B5	6585.03	6585.03 ± 90.45	2814.31	2814.31 ± 50.72	225	3.39182 × 10^−6^	significant

**Table 4:** Statistical analysis of the three different wire types using the Kruskal–Wallis test with a significance level of *p* < 0.001.

A5 Groups					
	Median				
Group I.A5	2468.46				
Group II.A5	3711.43				
Group III.A5	6585.03				
H = 39.130 with two degrees of freedom (*p* ≤ 0.001). All Pairwise Multiple Comparison Procedures (Tukey Test) *p*< 0.050:					
Comparison	Diff. of Ranks	q	*p*	*p* < 0.050	Interpretation
Group III.A5 vs. Group I.A5	450	8.847	<0.001	Yes	significant
Group III.A5 vs. Group II.A5	225	4.423	0.005	Yes	significant
Group II.A5 vs. Group I.A5	225	4.423	0.005	Yes	significant
**B5 Groups**					
	Median				
Group I.B5	1979.49				
Group II.B5	2400.38				
Group III.B5	2814.31				
H = 39.130 with two degrees of freedom (*p* ≤ 0.001). All Pairwise Multiple Comparison Procedures (Tukey Test) *p* < 0.050:					
Comparison	Diff. of Ranks	q	*p*	*p* < 0.050	Interpretation
Group III.B5 vs. Group I.B5	450	8.847	<0.001	Yes	significant
Group III.B5 vs. Group II.B5	225	4.423	0.005	Yes	significant
Group II.B5 vs. Group I.B5	225	4.423	0.005	Yes	significant

## Data Availability

The raw data supporting the conclusions of this article will be made available by the authors upon request.
